# Comparing the Effects of Different Cleansing Agents on the Shear Bond Strength of Resin Cements on Surface-Contaminated Zirconia: An In Vitro Study

**DOI:** 10.7759/cureus.78795

**Published:** 2025-02-09

**Authors:** Suriyanarayanan Karthikeyan, Kamala Kannan Raja, Balamurugan Anniyappan, Christina Dhivya Rani Soosairaj, Jawahar Lineous Devanbu Jayaseelan, Mukesh Kumar Srinivasan

**Affiliations:** 1 Department of Prosthodontics, Karpaga Vinayaga Institute of Dental Sciences, Chengalpet, IND

**Keywords:** hydrofluoric acid, monolithic zirconia, resin cements, shear bond strength, sodium hydroxide, universal testing machine

## Abstract

Background

The long-term success and durability of any dental ceramic material depends on how well they adhere to the resin cements. Though sandblasting of zirconia with abrasive particles is one of the most commonly used techniques to enhance the bond strength, surface damage and phase transition may affect the mechanical properties of zirconia. Hence alternative surface treatment methods using chemical agents, lasers, and silicone coatings can be employed.

Aim

To evaluate the shear bond strength (SBS) of two types of resin cement on surface-contaminated zirconia after using various cleaning agents.

Materials and methods

A total of 60 zirconia specimens were included in the study and divided into three groups, hydrofluoric (HF) acid, sodium hydroxide (NaOH), and a control, each containing 20 specimens. Within each group, the specimens were further subdivided into two subgroups based on the resin cement used: 10 specimens in the RelyX U200 subgroup (3M ESPE, St. Paul, MN) and 10 specimens in the Ivoclar SpeedCEM Plus subgroup (Ivoclar, Schaan, Liechtenstein). After surface treatment, the zirconia specimens were bonded to one of the two types of resin cement and subjected to a SBS test. A t-test was used to compare the resin cements, while an ANOVA test was conducted to compare the surface treatment groups.

Results

The NaOH group has shown significantly higher SBS in both RelyX U200 and Ivoclar SpeedCEM Plus subgroups than in the HF acid and control groups. RelyX U200 displayed superior adhesion to Ivoclar SpeedCEM Plus resin cement.

Conclusion

Surface treatment of zirconia with 5% NaOH exhibited increased SBS than HF acid-treated and non-surface-treated zirconia specimens. To strengthen the binding between zirconia and resin cements, NaOH may be used either on its own or in combination with other surface treatment methods.

## Introduction

All ceramic restorations such as glass-ceramics, polycrystalline, and resin matrix ceramics have surpassed the use of metal-ceramic restorations due to their biocompatible nature, superior esthetics, better optical properties, and minimal tooth preparation. Monolithic zirconia is a polycrystalline ceramic restorative material with high flexural strength (900 to 1200 MPa) and fracture toughness (9-10 MPa/m²) that has been widely used in the fabrication of crowns, short-span bridges, and veneers, inlays, and onlays, especially in patients with limited interocclusal space and parafunctional habits [1]. Shear bond strength (SBS) between zirconia and the luting agent is vital for the stability of the restoration. The SBS relies upon the cement used, application technique, and type of surface treatment done [2]. Resin-based-cements are more suitable for luting zirconia than conventional types of cement due to their superior marginal adaptation, durability, and increased fracture resistance [3]. The removal of bonding and etching chemicals has made self-adhesive resin cement the material of choice in recent years [4]. Porcelain, cast metal, composite, and endodontic post restorations have recently made use of self-adhesive resin cements such as RelyX U200 (3M ESPE, St. Paul, MN) and Ivoclar Speedcem Plus (Ivoclar, Schaan, Liechtenstein) for luting purposes.

A number of surface treatment methods were carried out to remove any contaminants from the zirconia and strengthen its binding to the resin cement. Sandblasting being the most commonly used surface treatment method, increases the roughness and surface energy of zirconia thus improving the adhesiveness of zirconia to resin cements [5,6]. Studies have found that zirconia to resin-based luting cements had an improved SBS after being sandblasted with abrasive particles [7]. Despite these advantages, surface damage and phase transition from tetragonal to monoclinic may have a negative impact on the mechanical properties and adhesiveness of zirconia [8]. 

Recently acid-based and alkaline-based chemicals have been used for the surface conditioning of ceramic restorations. Hydrofluoric acid (HF) of varying concentrations from 4-10% has been widely used as a surface conditioning agent [9]. Various studies have evaluated the effect of HF on surface modification of dental ceramics and concluded that HF creates surface roughness by reacting with the silica phase of the glass matrix to form hexafluorosilicates [10]. Other acids like phosphoric acid and nitric acid have also been experimented with for the surface cleansing of ceramic restorations [11,12]. Sodium hydroxide (NaOH) is an alkaline-based surface cleansing agent that has shown successful outcomes when used on glass ceramic restorations [13]. The impact of NaOH on zirconia and other ceramic restorations still requires more investigation. The purpose of this research is to find out how different chemical agents, such as 9% HF and 5% NaOH, influence the SBS of zirconia specimens attached to RelyX U200 and Ivoclar SpeedCEM plus composite resin cements using a universal testing machine.

## Materials and methods

Study design

The research was conducted inside the dental laboratory section of the Prosthodontics department at Karpaga Vinayaga Institute of Dental Sciences, India. The study received Institutional Ethics Committee (IEC) approval with a certificate number (KIDS/IEC/2024/II/009). The research utilized G* Power (version 3.1.9.4, Heinrich-Heine-Universität Düsseldorf, Düsseldorf, Germany) to determine 60 zirconia specimens as its sample with both 0.05 alpha error and 0.42 effect size. The power of the study was considered to be 0.80. After surface contamination, zirconia specimens were equally categorized into three groups such as HF acid group, NaOH group, and control group with 20 specimens in each group. These groups were further divided into two subgroups of 10 specimens each based on whether RelyX U200 or Ivoclar SpeedCEM Plus was used for bonding.

Fabrication of specimens and surface treatment

**Figure 1:**
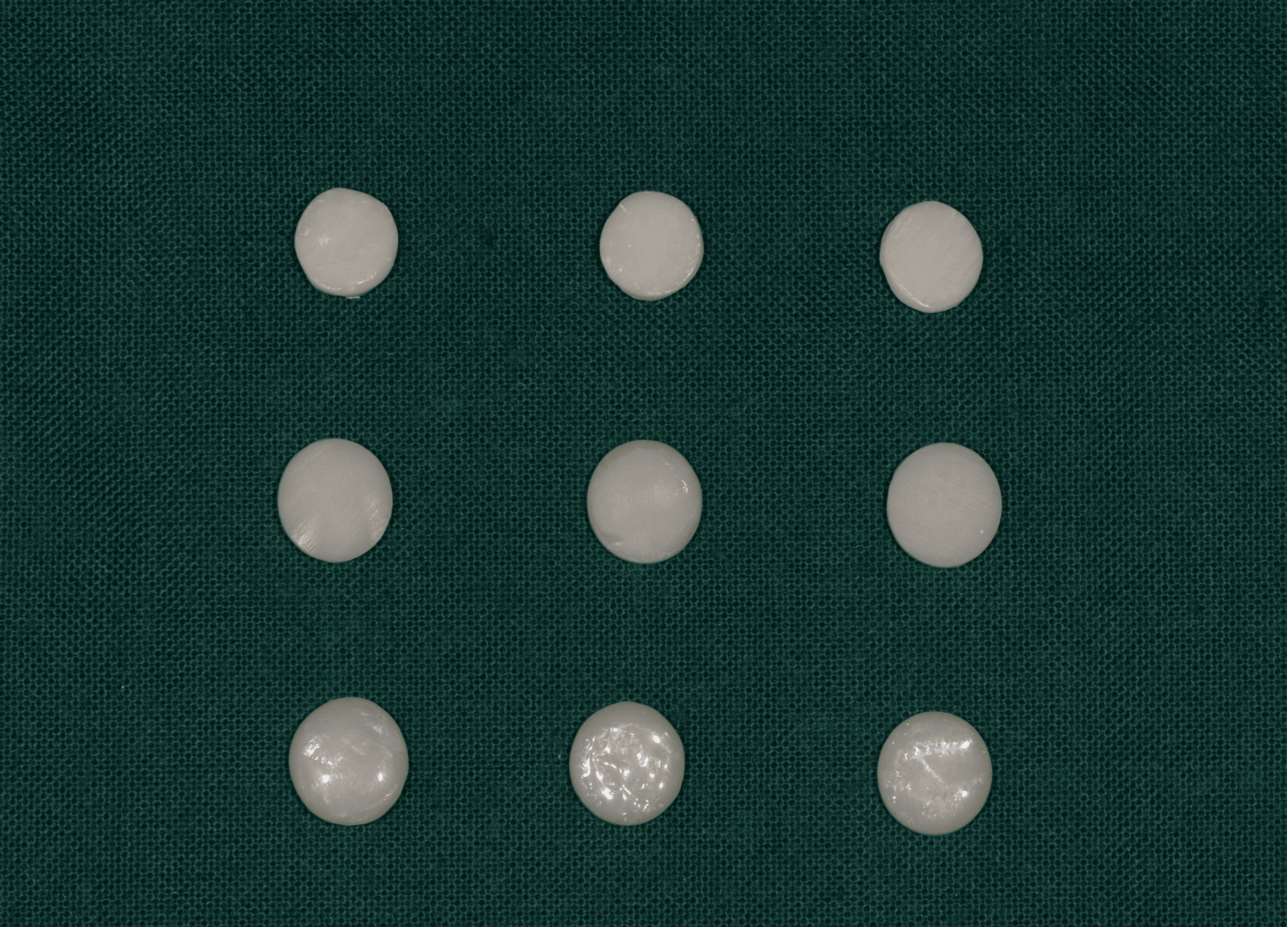
Disc-shaped zirconia specimens

**Figure 2:**
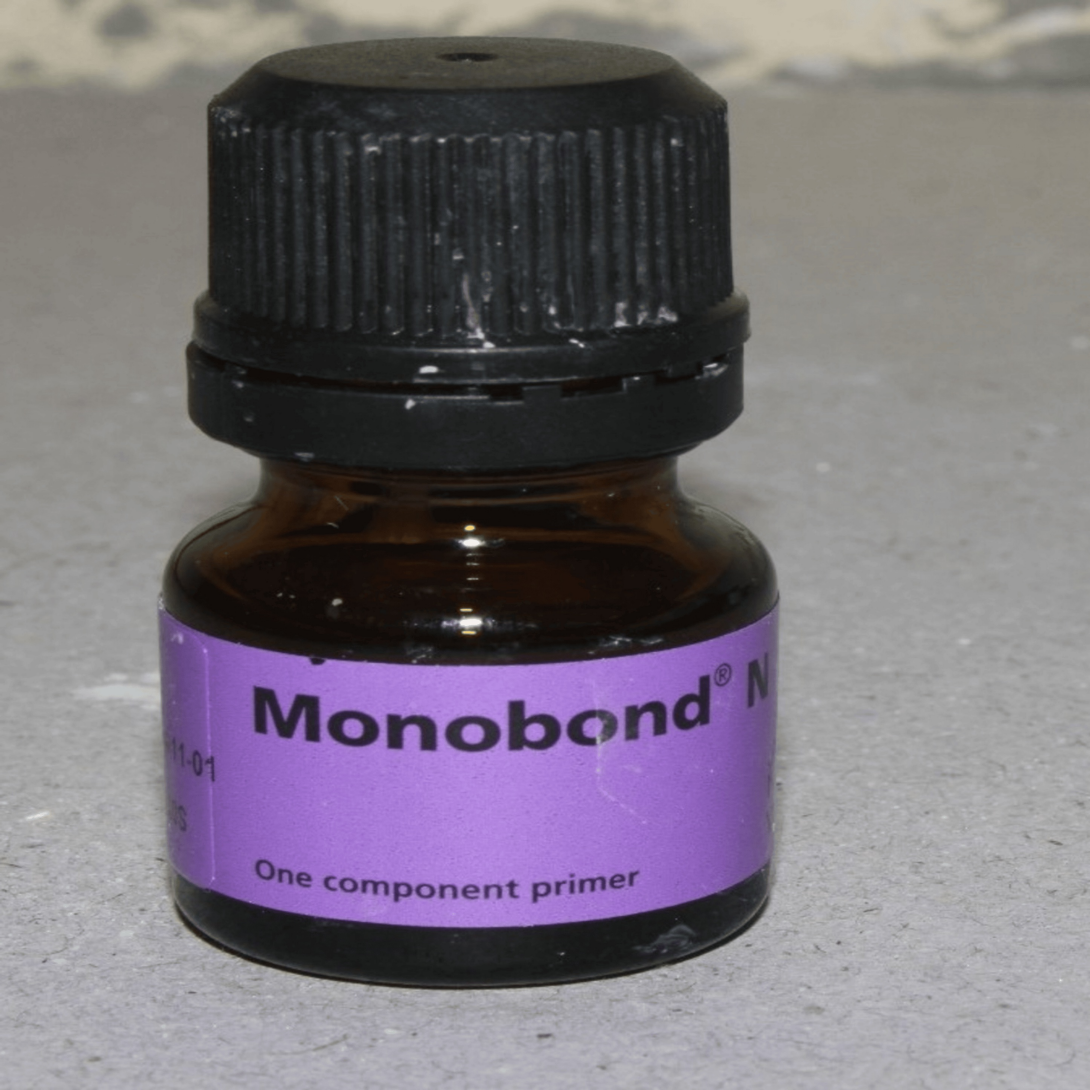
Priming agent used in the study Ivoclar Vivadent Monobond Plus primer

A total of 60 disc-shaped zirconia specimens of dimensions 10 x 2 mm were fabricated and sintered (Figure 1) by using VITA ZYRCOMAT 6000 MS (VITA Zahnfabrik H. Rauter, Bad Säckingen, Germany). The specimens were polished with silicone-coated carbide paper (1000 grit). Artificial saliva was sprayed over the specimens, which were then washed with water and dried thoroughly. In two groups, surface-contaminated zirconia specimens were subjected to surface treatment with 9% hydrofluoric acid (HF) gel and 5% sodium hydroxide (NaOH) solution for 20 seconds, respectively, while the control specimens were left without any surface treatment. The surface-treated specimens were washed with water. Ivoclar Vivadent Monobond Plus primer (Figure 2) was applied over all the zirconia specimens and left to react for 60 seconds.

Bonding of resin cements to study specimens

Polytetrafluoroethylene (PTFE) tubes were placed over each zirconia specimen with the following dimensions: 12 mm in height, 10 mm in inner diameter, and 12 mm in outer diameter. The PTFE tubes were filled with resin cement (RelyX U200 and Ivoclar SpeedCEM Plus) and cured for 40 seconds. The PTFE tubes were removed and the specimens were kept at 37˚ C in an incubator for 24 hours.

Determination of shear bond strength

**Figure 3:**
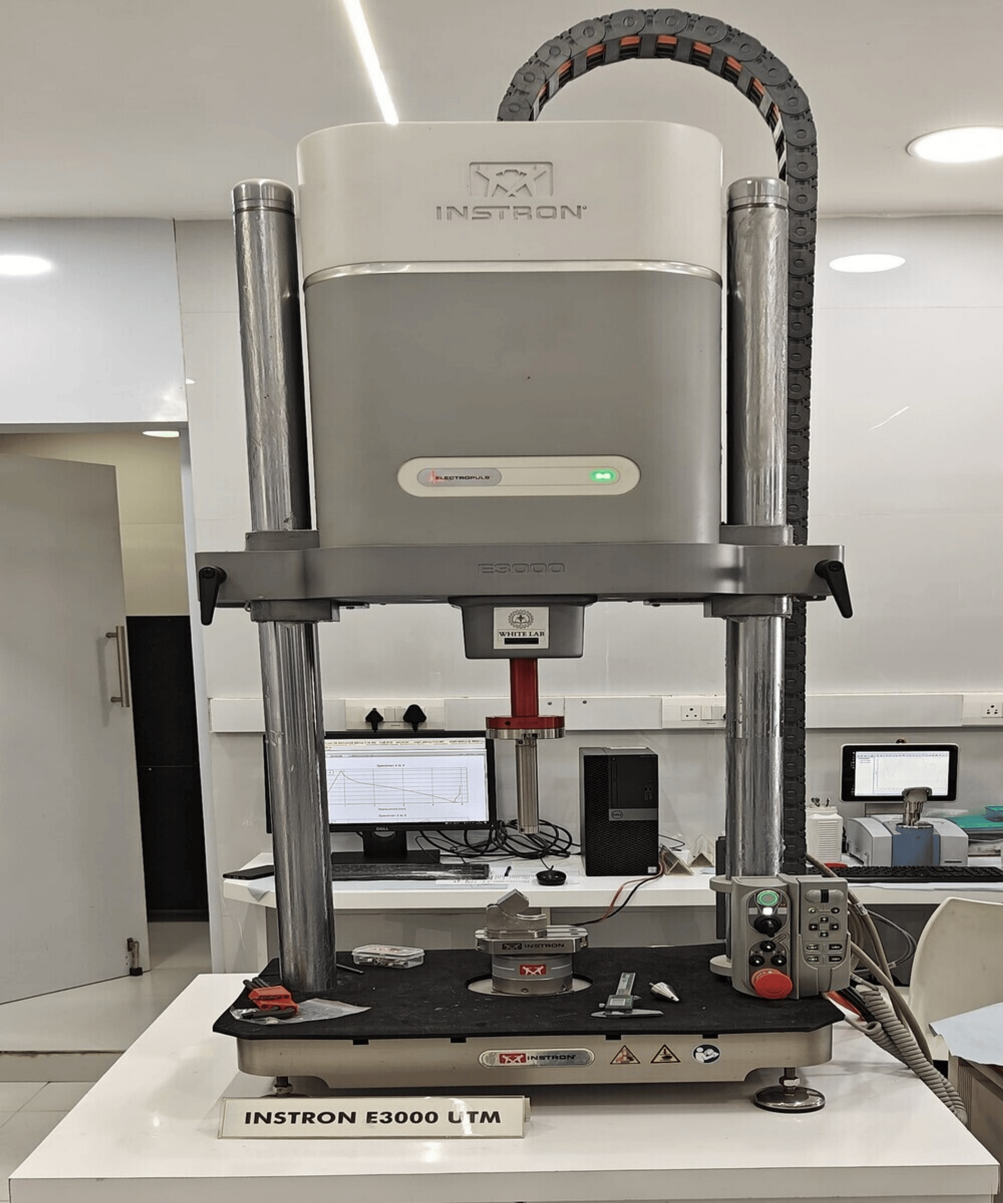
Universal testing machine ElectroPuls® E3000 Linear-Torsion: linear-torsion all-electric dynamic test instrument (Instron, Norwood, MA)

Figure 3 shows the SBS test performed on each zirconia specimen using a universal testing machine. The shear stress was automatically implemented at 0.5 mm/minute after placing the samples in position. The cross-sectional area (mm^2^) of each specimen and the force (in Newtons) were divided to get the SBS value in megapascals (MPa).

Statistical analysis

SPSS version 22.0 (IBM Corp., Armonk, NY) was used to calculate the mean SBS. A t-test was used for bond strength assessment of the two resin cements that received different surface treatments. Analysis of variance (ANOVA) examination and Tukey's Honestly Significant Difference post hoc test evaluated the bond strength of resin cements. A p-value under 0.05 marked statistical significance in this study.

## Results

Using the t-test, it was determined how surface treatment affected the SBS of composite resins when applied to zirconia specimens (Table 1). The mean SBS of RelyX U200 and Ivoclar SpeedCEM Plus composite resins after surface treatment with 9% HF was 10.52 ± 1.01 MPa and 7.55 ± 1.08 MPa respectively. After surface treatment with 5% NaOH, the mean SBS of RelyX U200 and Ivoclar SpeedCEM Plus was 21.86 ± 2.66 MPa and 15.67 ± 2.11 MPa respectively. A mean SBS of 5.51 ± 1.09 MPa and 3.99 ± 0.98 MPa was noted in RelyX U200 and Ivoclar SpeedCEM Plus subgroups after no surface treatment. Statistical analysis revealed that the two composite resins differed significantly in the HF and NaOH groups.

**Table 1:** SBS of composite resins to zirconia after different surface treatments (t-test results) A p-value less than 0.05 is considered statistically significant. HF acid: hydrofluoric acid; NaOH: sodium hydroxide; SBS: shear bonding strength; SD: standard deviation

Surface treatment groups	Composite resins (subgroups)	SBS mean ± SD (MPa)	t-value	p-value
HF acid group	RelyX U200	10.52 ± 1.01	6.312	0.001
	Ivoclar SpeedCEM Plus	7.55 ± 1.08		
NaOH group	RelyX U200	21.86 ± 2.66	5.76	0.001
	Ivoclar SpeedCEM Plus	15.67 ± 2.11		
Control group	RelyX U200	5.51 ± 1.09	3.25	0.005
	Ivoclar SpeedCEM Plus	3.99 ± 0.98		

**Figure 4:**
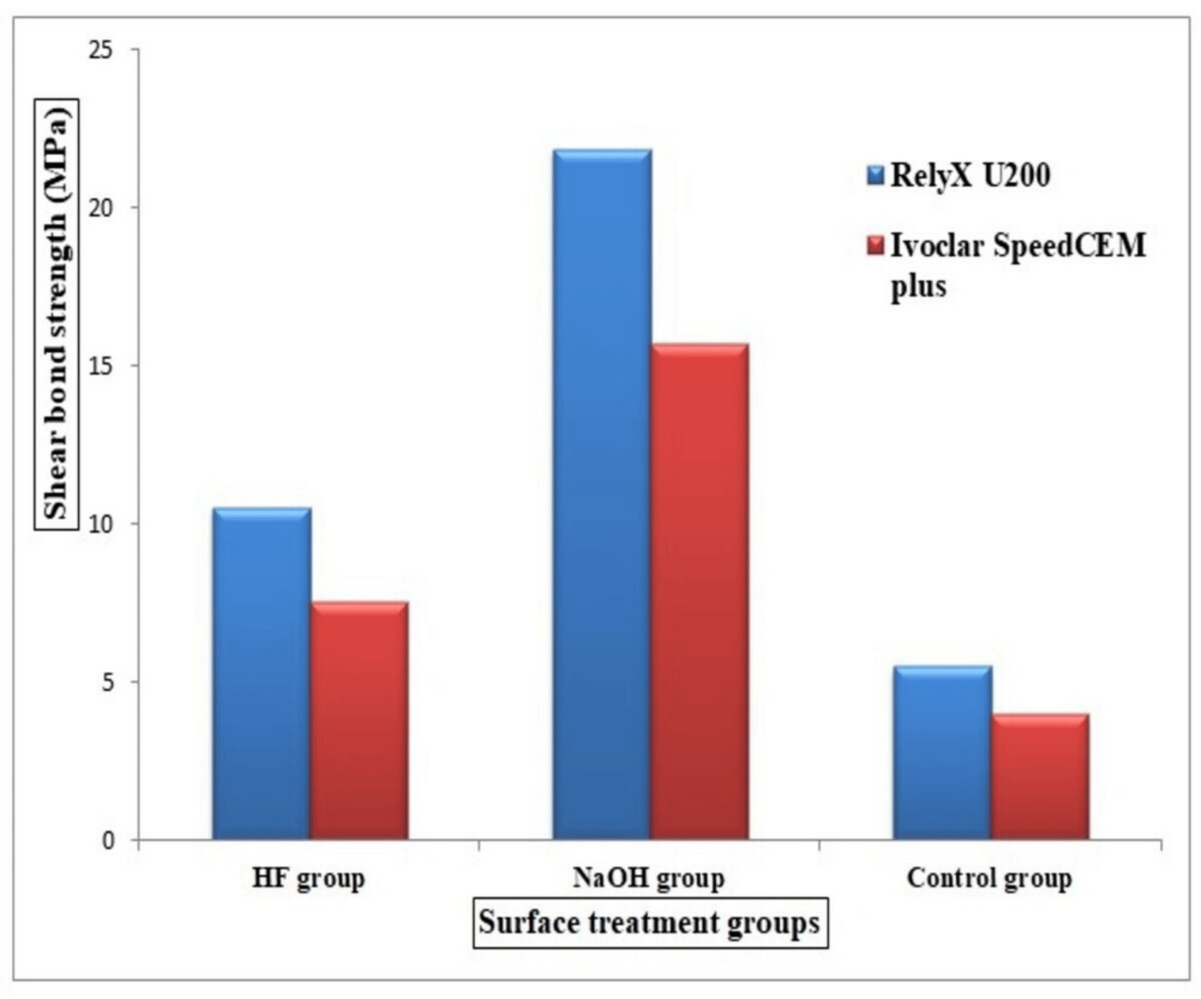
Comparison of surface treatment agents between the two composite resin subgroups HF: hydrofluoric acid; NaOH: sodium hydroxide

On comparing the surface treatment agents, the NaOH group showed significantly higher SBS in both RelyX U200 and Ivoclar SpeedCEM Plus subgroups than HF and no surface treatment groups. Table 2 reveals statistically significant differences in SBS between the three groups within the RelyX U200 and Ivoclar SpeedCEM Plus subgroups. Figure 4 denotes the comparison of surface treatment agents among the composite resin subgroups. A post hoc Tukey's HSD test established significant differences between results from the HF and NaOH groups and the untreated control group (Table 3).

**Table 2:** Comparison of surface treatment agents among the RelyX U200 and Ivoclar SpeedCEM Plus composite resin subgroups using ANOVA A p-value less than 0.05 is considered statistically significant. HF acid: hydrofluoric acid; NaOH: sodium hydroxide; SBS: shear bonding strength; SD: standard deviation

Composite resins (subgroups)	Surface treatment agents	SBS mean ± SD (MPa)	F-value	p-value
RelyX U200	HF acid	10.52 ± 1.01	226.05	0.001
	NaOH	21.86 ± 2.66		
	Control	5.51 ± 1.09		
Ivoclar SpeedCEM Plus	HF acid	7.55 ± 1.08	162.23	0.001
	NaOH	15.67 ± 2.11		
	Control	3.99 ± 0.98		

**Table 3:** Post-hoc Tukey's Honestly Significant Difference test comparing surface treatment agents across subgroups A p-value less than 0.05 is considered statistically significant. HF acid: hydrofluoric acid; NaOH: sodium hydroxide; SBS: shear bonding strength; SD: standard deviation

Composite resins (subgroups)	Comparison of surface treatment agents	SBS mean difference (MPa)	p-value
RelyX U200	HF vs NaOH	11.33	0.001*
	HF vs control	5.01	0.001*
	NaOH vs control	16.35	0.001*
Ivoclar SpeedCEM Plus	HF vs NaOH	8.11	0.001*
	HF vs control	3.56	0.001*
	NaOH vs control	11.67	0.001*

## Discussion

The clinical success and durability of any dental material depend on its biomechanical properties and adhesive nature. Improper adhesion or bonding of zirconia may lead to poor marginal adaptation and an increased chance of microleakage and fracture [14]. This is avoidable if the zirconia and luting cements can achieve their maximal bond strength. A combination of mechanical (air abrasion, lasers) and chemical surface treatments (acid/alkaline agents, silicone, and coupling agents) have been found to improve the adhesive properties of zirconia [15].

The current investigation included the surface treatment of monolithic zirconia specimens with various cleansing chemicals, including 9% HF and 5% NaOH. A universal testing machine was utilized to evaluate the SBS between RelyX U200 and Ivoclar SpeedCEM Plus resin cements and zirconia specimens. Since shear stresses are applied perpendicular to the bonded surfaces, SBS remains the most popular method for evaluating the strength of the adhesion between two dental materials [16].

HF acid works successfully as a surface treatment agent to enhance the resin cement-zirconia binding strength and on other dental ceramic restorations. Kim et al. concluded that 9% HF etching on zirconia specimens resulted in a significantly higher SBS than sandblasting and no surface treatment group [17]. In another study, the 10% HF showed a higher bond strength than other acids like 37% phosphoric acid, and citric acid [18]. In this present study, the HF surface treatment group had a significantly increased SBS value than the control group which was similar to the previous studies.

The surface treatment with 5% NaOH has shown an increased SBS than the HF acid and no surface treatment groups. Zavare et al. stated that alkali agents like NaOH and zirconium hydroxide when used as a surface treatment agent resulted in higher SBS between sandblasted zirconia specimens and composite resins. The possible mechanism behind this effect of NaOH may be due to the increase in hydroxide (OH) - groups and separation of hydrogen (H+) ions on the zirconia surface thereby leading to increased surface wettability [19]. In a comparative study conducted by Flores-Ferreyra et al., it was observed that HF acid had a decreased SBS value while the NaOH group had a superior SBS between zirconia and resin cements [20].

The shortcomings of the study include a small number of study specimens, no comparison with sandblasting and other mechanical surface treatments, and an inability to replicate temperature fluctuations and the oral environment. Further large-scale studies are needed to determine how variations in oral pH and temperature affect bonding strength between zirconia and resin cements.

## Conclusions

Surface treatment of monolithic zirconia with 5% NaOH (alkali-based) exhibits significantly higher shear bond strength than 9% HF acid (acid-based) surface cleansing agent. Additionally, RelyX U200 composite resin displayed better adhesion than Ivoclar SpeedCEM Plus in both surface-treated and non-surface-treated specimens. This suggests that alkali-based chemicals may be more effective in enhancing the bond strength between dental ceramics and resin-based luting cement variants, either alone or in combination with other surface treatment agents.
